# Relation of the Medical Profession to the Public

**Published:** 1860-07

**Authors:** 


### NEWS ITEMS, MEDICAL FACTS, &c.

(FROM THE "LONDON LANCET.")

*Relation of the Medical Profession to the Public.*—At a late meeting of the Royal Institution, Dr. Mayo delivered a lecture "On the Relation of the Public to the Science and Practice of Medicine." He alluded to an antagonistic feeling existing between medical men and a portion of the public, in consequence of which medical aid is often not applied for until it is too late. He recommended an alteration of the existing system, which tends to produce dissatisfaction and mutual distrust, and the adoption of a mode of practice that would place the public and the medical profession on terms of friendship. Dr. Mayo then strongly urged the propriety of a wider study of physiology as a branch of general education, and adverted favorably to the fitness of women to practice medicine. Touching on the subject of insanity, Dr. Mayo condemned the present system, which gives an arbitrary power of confinement to any two medical practitioners, who may or may not be competent to form an opinion as to insanity.

